# Cold-Induced Serum Short-Chain Fatty Acids Act as Markers of Brown Adipose Tissue Metabolism in Humans

**DOI:** 10.1210/clinem/dgaf607

**Published:** 2025-11-05

**Authors:** Milena Monfort-Pires, Mueez U-Din, Vanessa de Mello, Teemu Saari, Juho Raiko, Edla Kerminen, Johan Rajander, Kati Hanhineva, Tobias Fromme, Rikard Landberg, Martin Klingenspor, Kirsi A Virtanen

**Affiliations:** Turku PET Centre, University of Turku, Turku 20520, Finland; Turku PET Centre, Turku University Hospital, Turku 20520, Finland; Turku PET Centre, University of Turku, Turku 20520, Finland; Turku PET Centre, Turku University Hospital, Turku 20520, Finland; Institute of Public Health and Clinical Nutrition, School of Medicine, University of Eastern Finland, Kuopio 70211, Finland; Turku PET Centre, University of Turku, Turku 20520, Finland; Turku PET Centre, Turku University Hospital, Turku 20520, Finland; Turku PET Centre, Turku University Hospital, Turku 20520, Finland; Clinical Physiology and Nuclear Medicine Department, Kanta-Häme Central Hospital, Hämeenlinna 13530, Finland; Turku PET Centre, University of Turku, Turku 20520, Finland; Accelerator Laboratory, Turku PET Centre, Åbo Akademi University, Turku 20520, Finland; Department of Public Health and Clinical Nutrition, University of Eastern Finland, Kuopio 70211, Finland; Department of Life Technologies, Food Science Unit, University of Turku, Turku 20500, Finland; Department of Biology and Biological Engineering, Division of Food and Nutrition Science, Chalmers University of Technology, Gothenburg 41296, Sweden; Chair of Molecular Nutritional Medicine, TUM School of Life Sciences, Technical University of Munich, Freising 85354, Germany; EKFZ-Else Kröner Fresenius Center for Nutritional Medicine, Technical University of Munich, Freising 85354, Germany; Department of Life Sciences, Division of Food and Nutrition Science, Chalmers University of Technology, Gothenburg 41296, Sweden; Chair for Molecular Nutritional Medicine, Technical University of Munich, Freising 85354, Germany; EKFZ-Else Kröner Fresenius Center for Nutritional Medicine, Technical University of Munich, Freising 85354, Germany; ZIEL-Institute for Food & Health, Technical University of Munich, Freising 85354, Germany; Turku PET Centre, University of Turku, Turku 20520, Finland; Turku PET Centre, Turku University Hospital, Turku 20520, Finland

**Keywords:** short-chain fatty acids, brown adipose tissue, cold exposure, PET/CT scan

## Abstract

**Context:**

Short-chain fatty acids (SCFAs) produced from dietary fiber fermentation can regulate adipose tissue metabolism through signaling pathways involving G protein–coupled receptors and histone deacetylase inhibition. While preclinical studies suggest they enhance thermogenesis, their role in human brown adipose tissue (BAT) under different thermal conditions remains unclear.

**Objective:**

This study explores the associations between circulating SCFAs and human BAT metabolism at room temperature and after cold exposure.

**Methods:**

This cross-sectional study included data from 71 adults (aged 20-55 years, body mass index 19-44 kg/m^2^). Dynamic [^15^O]O_2_, [^15^O]H_2_O, [¹⁸F]FDG, and [¹⁸F]FTHA positron emission tomography/computed tomography scans were used to assess BAT metabolism. Serum SCFAs were quantified using liquid chromatography–mass spectrometry, and gene expression in biopsy-excised BAT samples (n = 14) was analyzed. Participants were stratified into low- and high-BAT groups based on [¹⁸F]FDG or [¹⁸F]FTHA uptakes.

**Results:**

Cold-induced acetate and propionate were positively associated with key in vivo BAT metabolism indicators, namely nonesterified fatty acid (NEFA) uptake and oxygen consumption. Only in the high-BAT group were circulating SCFAs maintained after cold exposure. BAT transcriptome revealed that genes involved in SCFA metabolism (such as conversion to acetyl-CoA) correlated with thermogenic and lipid metabolism genes exclusively in the high-BAT group, suggesting a distinct molecular link between SCFA pathways and BAT function.

**Conclusion:**

Circulating SCFAs are linked with BAT oxidative metabolism and NEFA uptake during cold exposure. The observed correlations between SCFA catabolic genes and thermogenic markers suggest that metabolically active BAT may selectively engage SCFA-related pathways, pointing to a potential mechanistic role of SCFAs in supporting BAT function in humans.

It has long been known that fiber intake is associated with favorable metabolic outcomes (1, 2), though only recently have some of these effects been linked to the actions of short-chain fatty acids (SCFAs) (2, 3). Acetate, propionate, and butyrate are produced in the colon by various bacterial families through the fermentation of dietary fiber, including resistant starch and, to a lesser extent, proteins (3-5). Although less than 10% of these SCFAs enter systemic circulation, they can still act as substrates or signaling molecules regulating diverse metabolic processes (3, 5).

The beneficial effects of SCFAs are shown to be primarily mediated by SCFAs functioning as ligands for G protein–coupled receptors (GPCRs), particularly GPR43 and GPR41 (3, 6, 7). Among them, there is evidence that butyrate can enhance whole-body insulin sensitivity by promoting glucose uptake in muscle and adipose tissue and suppressing hepatic glucose production, thereby supporting glycemic control (8). Acetate, conversely, serves as a substrate for cytosolic acetyl-CoA synthesis, contributing to lipogenesis and other acetyl-CoA–dependent processes critical for adipose tissue metabolism and function (9). Emerging evidence points to the fact that butyrate and acetate influence the secretion of gut-derived hormones such as peptide YY (PYY) and glucagon-like peptide-1 (GLP-1) by activating GPCRs (3, 6, 10, 11). These SCFAs also act as signaling molecules, exerting systemic effects across various tissues (12). Notably, acetate has been linked to reduced food intake and suppressed orexin neuronal activity in ob/ob mice (13). Beyond metabolic regulation, SCFAs are linked to anti-inflammatory effects, as they can inhibit the production of proinflammatory cytokines and reduce systemic inflammation (6, 14). These immunomodulatory actions are thought to be mediated in part by SCFA-induced inhibition of histone deacetylases (HDACs), particularly via GPR41 and GPR43 signaling—a mechanism considered central to the regulatory role of SCFAs (6, 11, 15).

Unlike white adipose tissue (WAT), which primarily serves as an energy reservoir, brown adipose tissue (BAT) dissipates energy as heat, positioning it as a promising target for cardiometabolic disease treatments (16-19). While the interaction between SCFAs and BAT function in humans remains underexplored, SCFAs are known to modulate adipose tissue metabolism by regulating lipolysis and lipogenesis (2, 5, 20). In vitro studies indicate that butyrate and propionate can induce WAT browning, enhance mitochondrial function, and boost energy expenditure via activation of adenosine monophosphate–activated protein kinase (AMPK) and peroxisome proliferator–activated receptor γ (PPAR-γ) pathways (21-23). The thermogenic effects of SCFAs also occur via the sympathetic nervous system, which leads to enhanced WAT browning and nonshivering thermogenesis (5, 8). Some studies show that SCFAs can also inhibit adipogenesis by suppressing lipogenic enzymes and promoting lipolysis, thereby preventing excessive fat accumulation (14, 24). Additionally, experimental data indicate that acetate and butyrate enhance BAT activity by upregulating thermogenic genes such as *UCP1* (5, 25), alongside improvements in overall energy metabolism by increasing fatty acid oxidation in BAT (15). Indeed, in rodents, acetate injections have been shown to reduce lipid accumulation in BAT while increasing the expression of lipolytic genes (25).

SCFAs exert beneficial metabolic effects through mechanisms involving the activation of GPCRs in different tissues (3, 6, 10). Rodent studies further highlight the crucial role of SCFAs in regulating WAT and BAT metabolism (5, 8, 25). However, the interaction between SCFAs and BAT, particularly under cold exposure and thermoneutrality conditions, remains poorly understood in humans. To address this gap, the present study investigated the associations between circulating SCFAs—acetate, propionate, and butyrate—and BAT metabolism in adult humans at room temperature (RT) and after acute cold exposure. Even though there are no standard thresholds to classify individuals as low or high BAT, we stratified our sample into low and high BAT to better understand how having more active BAT affects circulating SCFAs and gene expression profiles. Lastly, we explored the correlations between genes involved in SCFA signaling pathways (such as components of the SCFA utilization pathway, encompassing SCFA transport, mitochondrial conversion, and downstream metabolic regulation in BAT) and those regulating thermogenesis, lipolysis, and lipogenesis in BAT.

## Material and Methods

### Study Design and Participants

These cross-sectional data included 71 individuals from studies conducted at the Turku PET Centre between 2009 and 2018 (26-29). Imaging and biological data are derived from previous studies investigating human BAT metabolism. These cohorts’ inclusion and exclusion criteria have been previously published (27, 29).

Twenty-two men and 49 women were included in this study. The group had an age range of 20 to 55 years and a body mass index (BMI) range of 18.9-43.7 kg/m^2^. All participants were clinically assessed before recruitment into the respective studies, and those presenting with an overall healthy metabolic profile with no diabetes and/or cardiovascular diseases were included. A metabolic health profile was assessed and determined based on available medical records, a 2-hour oral glucose tolerance test, an electrocardiography assessment, circulating lipids, and hepatic enzymes profile. All recruited study individuals provided written informed consent for volunteering in the clinical research studies. The Hospital District of Southwest Finland Ethics Committee gave their favorable opinion for the study protocols, and the studies were conducted according to the principles of the Declaration of Helsinki (27-29).

All positron emission tomography/computed tomography (PET/CT) imaging and blood samples were collected after an overnight fast. Participants were instructed to refrain from consuming alcoholic and caffeinated beverages for 12 hours before the metabolic assessments. Additionally, participants were advised to avoid strenuous physical activity for 24 hours before the study.

### Biochemical Data

As previously described, fasting blood samples were used for biochemical analyses to measure plasma total cholesterol, high-density lipoprotein (HDL), and triglycerides (TGs) photometrically (ModularP800, Roche Diagnostics). Low-density lipoprotein (LDL) cholesterol was calculated using the Friedewald equation. Whole-body insulin sensitivity was assessed using hyperinsulinemic-euglycemic clamp (M-value) (27-29).

### Positron Emission Tomography/Computed Tomography Imaging

The scanning protocol was conducted during cold exposure using a personalized cooling protocol for both studies: For study 1, on the cold exposure day, the participants spent 2 hours wearing light clothing in a room with an ambient temperature of 17 °C before the PET imaging. During the imaging (ambient temperature of 23 °C), cold exposure was induced by placing 1 foot intermittently (5 minutes in/5 minutes out) in water at a temperature of 8 °C on average (26). For study 2, the cooling was 2 hours before the PET scan using cooling blankets, 6 °C water, and then increased water temperature when shivering was observed (objective observation by a researcher). During the imaging, the cooling protocol was maintained (27).

The PET imaging of the supraclavicular region was carried out while the participant was in a supine position. For study 1, the glucose uptake rate in the target tissues (BAT, WAT) was measured using the [^18^F]-FDG ([18F]2-fluoro-2- deoxy-D-glucose) radiotracer (μmol/100 g/min). For study 2, the nonesterified fatty acid (NEFA) uptake rate (μmol/100 g/min) was analyzed using the [^18^F]-FTHA (14(R,S)-[18F]fluoro-6-thia-heptadecanoic acid) radiotracer. Tissue perfusion at RT and after cold exposure was assessed using [^15^O]H_2_O (mL/100 g/min), whereas metabolic rate of oxygen (MRO_2_) was analyzed using [^15^O]O_2_ (mL/100 g/min) (27). As previously described, BAT radiodensity (measured with Hounsfield units, HU) was analyzed using CT images (30). All PET scans were performed using a dynamic acquisition protocol as previously described (27, 30).

### Positron Emission Tomography/Computed Tomography Imaging Analysis

PET/CT imaging data were analyzed using Carimas software (version 2.8, Turku PET Centre) and Vinci 2.54.0 software (Max-Planck Institute). Glucose and NEFA uptake were analyzed using the Patlak-Gjedde plot (31, 32). Participants were categorized as high BAT or low BAT based on substrate uptake rates ([^18^F]-FDG and/or [^18^F]-FTHA) in supraclavicular BAT depot. Specifically, individuals were classified as high-BAT if they had a glucose uptake rate greater than or equal to 3.0 μmol/100 g/min or an NEFA uptake rate greater than or equal to 0.7 μmol/100 g/min. The threshold for the glucose uptake rate of 3.0 μmol/100 g/min is based on a 3-fold increase during cold exposure compared to resting, RT measurements (1.0 μmol/100 g/min) (29). The threshold aligns with a standardized uptake value (SUV) of 1.5 g/mL (30), the recommended SUV for BAT detection according to the BARCIST 1.0 criteria (33). The NEFA uptake rate of 0.7 μmol/100 g/min is equivalent to the energy content of glucose following complete oxidation.

### White and Brown Adipose Tissue Biopsies and RNA Sequencing

The biopsy of supraclavicular adipose tissue for WAT and BAT was taken from 14 volunteers (30). BAT depots in this region were identified using available imaging data, such as magnetic resonance imaging or cold-exposed [^18^F]-FDG PET and [^18^F]-FTHA PET/CT scans. A subcutaneous adipose tissue sample was collected from the same incision site as a WAT reference. The procedure was conducted at RT (∼22 °C) under local anesthesia with lidocaine-epinephrine and performed by a plastic surgeon. Immediately after extraction, tissue samples were frozen in liquid nitrogen to preserve their integrity.

Deep-frozen adipose tissues (30-120 mg) were homogenized in TRIsure, and RNA was extracted, purified using spin columns, and eluted in water. RNA concentration was measured (Nanodrop ND-1000), diluted (25-500 ng/mL), denatured (70 °C, 2 minutes), and assessed for integrity (Bioanalyzer 2100). Coding RNA was enriched (TruSeq RNA Access) to compensate for degradation, and sequencing was performed (HiSeq 2500, Illumina). Gene expression levels were quantified as reads per kilobase per million reads (27, 28).

### Short-Chain Fatty Acid Quantification

Targeted quantification of SCFAs (acetate, propionate, butyrate) was performed using a liquid chromatography–mass spectrometry method developed and validated at Chalmers University of Technology (34). Standards were sourced from Honeywell (acetate), Alfa Aesar (propionate), and Sigma Aldrich (butyrate). Derivatization reagents (3-NPH, EDC-6, quinic acid, pyridine, methanol, water) were from Sigma Aldrich; acetonitrile was from Fisher Scientific. A ^13^C₆-3NPH-HCl internal standard, synthesized by IsoSciences Inc, was used for quantification. Stock solutions of standards were prepared in 75% methanol at specified concentrations and diluted 1:10 in 10% methanol. Calibration curves ranged 3.2 µM to 0.63 nM (acetate), 3.2 µM to 0.31 nM (propionate), and 0.8 µM to 0.16 nM (butyrate). Samples were analyzed in batches, and each batch included 5 blanks and 3 triplicate quality control samples (2 long-term pooled samples; 1 cohort-specific sample).

### Statistical Analysis

All statistical analyses were performed using IBM SPSS Statistics 28.0.1. Figures were drawn using GraphPad Prism version 10.0 (Graphpad Software) and Python version 3.4 (Python Software Foundation). Data are presented as mean and SD or SEM. The normality of data was checked using the Kolmogorov-Smirnov test. Because most variables were not normally distributed, Spearman correlation coefficients were used for correlation analyses, and a false discovery rate (FDR) test for multiple comparisons was applied (correlation heat maps). For the correlations between circulating SCFAs and imaging parameters, the variables were log-transformed, and the Pearson coefficient was employed. Low-BAT and high-BAT correlation coefficients were compared using the Fisher r-to-z test. An independent *t* test (or Mann-Whitney *U*) was also used to compare low-BAT and high-BAT groups. For paired analysis (WAT vs BAT gene expression in the same group of individuals), a paired-sample *t* test (or Wilcoxon) was employed.

## Results

### Characteristics of the Sample

The main characteristics of the sample are presented in Table 1. As previously mentioned, the cutoff used to categorize volunteers as low- and high-BAT aligns with an SUV of 1.5 g/mL, and its application allowed for the detection of significant group differences, supporting its utility in this context. Therefore, Table 1 shows the volunteers’ baseline characteristics according to low- or high-BAT groups. Predictably, the high-BAT group consisted of younger, leaner individuals with better metabolic profiles, such as lower concentrations of total and LDL cholesterol and TGs, higher HDL levels, and higher insulin sensitivity, as assessed by the M-value (*P* < .05 for all). Only body fat percentage was similar between the groups. It is important to emphasize that these data have been previously shown by our group (30).

**Table 1.** Clinical data and circulating short-chain fatty acids in low–brown adipose tissue (BAT) and high-BAT individuals

	Low-BAT (n = 32)	High-BAT (n = 39)	*P*	Total sample
**Clinical data**				
Female participants, n; %	17 (53%)	32 (82%)	<.01	49 (69%)
Age, y*a*	43.7 ± 7.5	35.9 ± 10.7	<.01	39.4 ± 10.1
Weight, kg	88.7 ± 19.1	70.6 ± 12.4	<.01	78.7 ± 18.1
BMI (kg/m^2^)	29.8 ± 6.5	24.8 ± 3.8	<.01	27.1 ± 5.7
Body fat, %	33.5 ± 11.3	31.8 ± 8.1	.46	32.5 ± 9.6
Waist circumference, cm	98.2 ± 18.7	84.2 ± 12.9	<.01	90.7 ± 17.2
Hip circumference, cm*a*	106.6 ± 14.3	97.9 ± 9.8	<.01	101.9 ± 12.8
Insulin sensitivity, M-value, µmol/kg/min	30.3 ± 17.7	47.1 ± 20.2	<.01	39.8 ± 20.8
HDL-c, mmol/L	1.5 ± 0.3	1.7 ± 0.4	.04	1.6 ± 0.4
Total cholesterol, mmol/L	5.0 ± 0.8	4.4 ± 0.9	<.01	4.7 ± 0.9
LDL-c, mmol/L*a*	3.0 ± 0.8	2.5 ± 0.7	<.01	2.7 ± 0.8
TGs, mmol/L*a*	1.2 ± 0.7	0.8 ± 0.3	<.01	1.0 ± 0.5
**Serum SCFAs, RT (n = 33)**				
Fasting acetate, µM*a*	103.2 ± 63.0	125.6 ± 75.5	.47	124.7 ± 84.0
Fasting propionate, µM*a*	1.4 ± 0.6	1.5 ± 0.8	.78	1.4 ± 0.7
Fasting butyrate, µM*a*	0.4 ± 0.3	0.4 ± 0.17	.37	0.4 ± 0.2
**Serum SCFAs, CI (n = 72)**				
Fasting acetate, µM*a*	91.1 ± 50.7	121.3 ± 80.0	.06	106.0 ± 69.3
Fasting propionate, µM	0.9 ± 0.5	1.2 ± 0.6	.02	1.1 ± 0.5
Fasting butyrate, µM*a*	0.2 ± 0.1	0.4 ± 0.4	.13	0.3 ± 0.3
**BAT-imaging data, RT**				
Glucose uptake rate, μmol/100 g/min	1.0 ± 0.2	1.0 ± 0.6	.48	1.0 ± 0.4
NEFA uptake rate, μmol/100 g/min	0.3 ± 0.2	0.9 ± 0.7	<.01	0.6 ± 0.6
Perfusion, mL/100 g/min	4.9 ± 3.7	8.1 ± 4.0	.02	6.7 ± 4.1
MRO_2_, mL/100 g/min	0.5 ± 0.1	0.7 ± 0.1	.13	0.6 ± 0.1
**BAT-imaging data after CI**				
Glucose uptake rate, μmol/100 g/min	1.1 ± 0.6	8.1 ± 5.5	<.01	4.9 ± 5.3
NEFA uptake, μmol/100 g/min	0.4 ± 0.1	1.4 ± 1.1	<.01	1.0 ± 0.9
Perfusion, mL/100 g/min	9.8 ± 4.0	14.8 ± 6.6	<.01	12.9 ± 6.2
MRO_2_, mL/100 g/min	1.3 ± 0.4	1.8 ± 0.6	.076	1.6 ± 0.6

Abbreviations: BAT, brown adipose tissue; BMI, body mass index; CI, cold-induced; HDL, high-density lipoprotein; LDL, low-density lipoprotein; MRO_2_, metabolic rate of oxygen; NEFA, nonesterified fatty acids; RT, room temperature; SCFA, short-chain fatty acid; TGs, triglycerides.

Independent-samples *t* test/*^a^*nonparametric test (Mann-Whitney *U*).

No differences between the two groups were detected regarding circulating SCFAs at RT. However, we observed higher concentrations of cold-induced (CI) propionate (*P* = .02) and a trend of higher concentrations of CI-acetate in the high-BAT group when compared to low-BAT (*P* = .06), suggesting that these SCFAs could be modulated by acute cold exposure. Differences in BAT-imaging data between groups were expected because we used CI-NEFA uptake and glucose uptake rate (GUR) to stratify the groups. Still, no differences were detected for GUR and MRO_2_ at RT and CI-MRO_2_ (*P* = .078), most likely due to the small sample size for these measurements.

### Associations Between Imaging Data, Circulating Cardiometabolic Markers, and Short-Chain Fatty Acids

**Figure 1.**
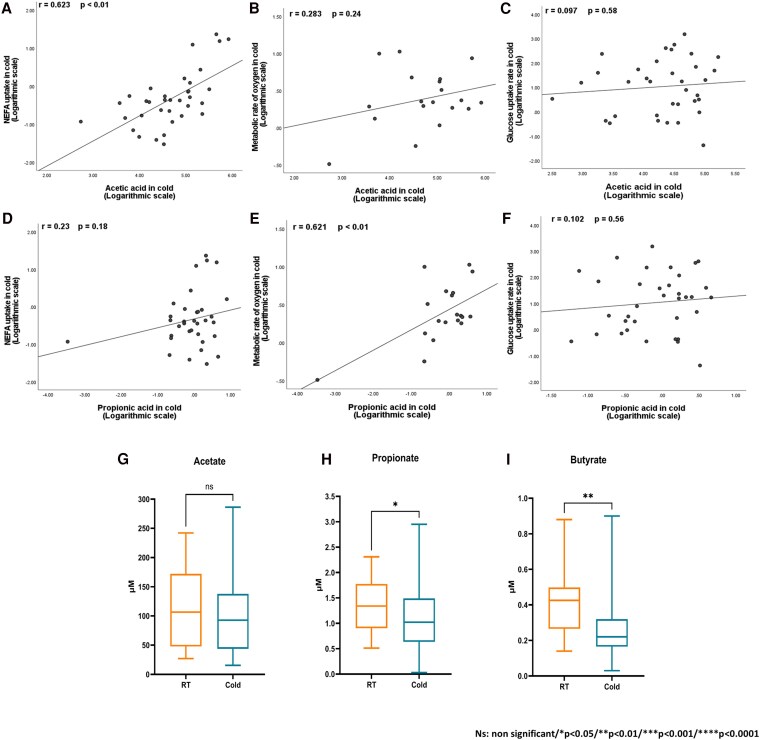
Correlations between A, nonesterified fatty acid (NEFA) uptake; B, metabolic rate of oxygen; and C, glucose uptake rate in cold-stimulated brown adipose tissue (BAT) with cold circulating acetate, and propionate (D, E, F respectively, log-transformed data), and G, circulating acetate; H, propionate; I, and butyrate at room temperature (RT) and after 2 hours of cold exposure (cold-induced). Sample sizes differed for different tracers. Cold-induced NEFA uptake (A and D; n = 35); metabolic rate of oxygen (B and E; n = 19), and glucose uptake rate (C and F; n = 35). For the comparisons between RT and CI-SCFA (panels G-I) n = 33.

Next, we sought to understand how SCFAs were associated with BAT function. Therefore, we analyzed the correlations between RT and CI-SCFAs with BAT variables (GUR, NEFA uptake rate, and MRO_2_ in BAT) under RT and CI conditions (Fig. 1 and Supplementary Table S1) (35). Interestingly, CI-acetate was directly correlated with CI-NEFA uptake (*r* = 0.62; *P* < .01), whereas CI-propionic acid was associated with the CI-MRO_2_ (*r* = 0.62; *P* < .01). Moreover, CI-acetate correlated with RT and CI circulating free fatty acids (CI-FFAs) (*P* < .01; see Supplementary Table S1) (35). In contrast, RT-acetate was associated with CI-FFA, BAT radiodensity, and CI-BAT NEFA uptake (see Supplementary Table S1) (35). Nevertheless, after FDR correction, only the associations between FFAs and acetate remained statistically significant (see Supplementary Table S1) (35). In addition, RT and CI-butyrate were associated with BAT radiodensity. In contrast, CI-acetate concentrations were inversely correlated with clinical markers such as body weight, BMI, and waist and hip circumference. At the same time, RT acetate was associated with M-value (*P* < .01; not statistically significant after FDR correction). RT and CI-butyrate were also inversely correlated with waist circumference, while CI-butyrate was also associated with M-value (see Supplementary Table S1) (35).

### Changes in Acetate, Butyrate, and Propionate After Cold Exposure

Because we observed correlations between CI-SCFA and imaging data and cold stress could affect SCFA levels (18, 36), we investigated how acetate, propionate, and butyrate levels were modulated during acute cold exposure. We observed that propionate and butyrate levels were lower after cold exposure than RT (Fig. 1H and 1I), but no statistically significant changes were detected in acetate concentrations (Fig. 1G).

### Differences Between Low–Brown Adipose Tissue and High–Brown Adipose Tissue Groups

**Figure 2.**
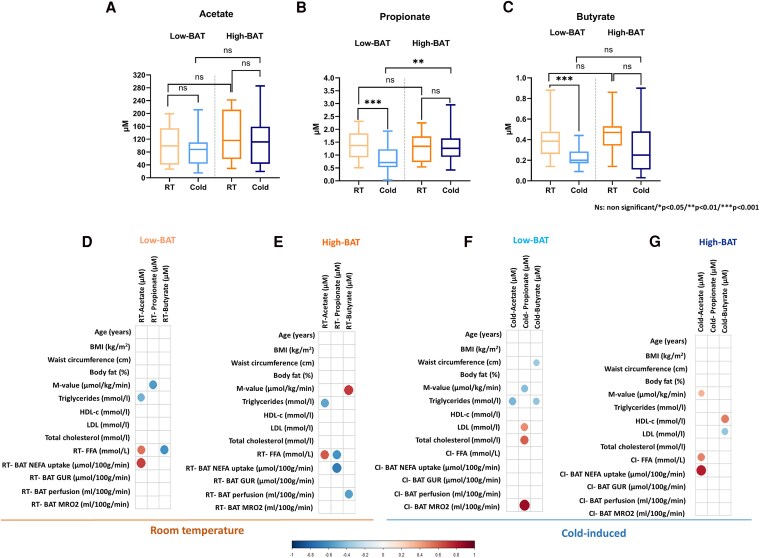
Circulating A, acetate; B, propionate; and C, butyrate at room temperature (RT) and after 2 hours of cold exposure in low- vs high–brown adipose tissue (BAT) volunteers and Spearman correlations between D and E, circulating room temperature and F and G, cold-induced short-chain fatty acids (SCFAs) with clinical variables. Bigger circles indicate stronger correlation analysis or lower *P* values. Circulating SCFAs (RT: n = 33, CI: n = 72). RT-BAT NEFA uptake (n = 18), RT-BAT glucose uptake (n = 18), RT-BAT perfusion (n = 25), RT-BAT metabolic rate of oxygen (MRO_2_) (n = 6), CI-BAT NEFA uptake (n = 35), CI-BAT glucose uptake (n = 35), CI-BAT perfusion (n = 54), CI-BAT MRO_2_ (n = 19).

To better understand how changes in SCFAs were affected by having a higher or lower BAT metabolism, we investigated whether CI changes in SCFA were different when comparing low-BAT and high-BAT volunteers. Even though acetate concentrations were similar between low- and high-BAT groups (Fig. 2A), in the high-BAT group, propionate and butyrate levels were maintained after 2 hours of cold exposure (Fig. 2B and 2C). We observed reductions in serum propionate (Fig. 2B) and butyrate (Fig. 2C) after cold exposure only in the low-BAT group. Moreover, the low-BAT group showed lower propionate concentrations after cold exposure (see Fig. 2B).

Differences between the low-BAT and high-BAT groups were also observed when analyzing the correlations between SCFA and clinical variables (Fig. 2D-2G and Supplementary Table S2) (35). Positive associations between BAT NEFA uptake and CI-acetate were detected only in the high-BAT group (ρ = 0.781; *P* < .01) (see Fig. 2G), whereas CI-propionate was associated with the MRO_2_ in BAT (ρ = 0.857; *P* < .05) only in the low-BAT group (see Fig. 2F). On the other hand, RT propionate and butyrate were inversely associated with CI-NEFA uptake in the low-BAT but not in the high-BAT group (see Fig. 2D and 2E). A statistically significant association between CI-acetate and CI-FFAs was found only for the latter group (Fig. 2G). Concerning clinical variables, RT-butyrate correlated with M-value (ρ = 0.703; *P* < .01) and CI-butyrate with HDL only in the high-BAT group. Conversely, RT-acetate was inversely correlated with body weight, while CI-propionate was associated with LDL and total cholesterol in the low-BAT group (see Fig. 2D-2G and Supplementary Table S2) (35).

### Gene Expression Associations Between Short-Chain Fatty Acid Metabolic Pathways With β-Oxidation, De Novo Lipogenesis, and Thermogenic Markers

Because SCFAs have been shown to act as substrates for BAT function via de novo lipogenesis (DNL), converted into acetyl-CoA for the tricarboxylic acid cycle (TCA) during acute cold exposure, and influence HDACs, we sought to investigate how thermogenic genes were correlated to SCFA metabolic pathway (ie, enzymes promoting DNL from SCFAs and catabolic pathways) gene expression in BAT samples. When we analyzed the entire sample (Supplementary Fig. S1) (35), we observed strong associations between *ACSS1* with *SIRT3* and *ACSS3* with *HADH*, *FASN*, and *CEBPA*. Other genes associated with the SCFA catabolic pathways (*PCCA*, *PCCB*, *MUT*, *HADH*, *HADHA*, *HADHB*, and *ECSH1*) were correlated both to β-oxidation genes (such as *AKT2*, *CPT2*, and *PRKAKA*) and to the genes in the DNL/FA synthesis pathways (*ACACA*, *ACLY*, *ELOVL6*). In addition, these genes showed strong positive associations with thermogenic markers (*UCP1*, *CIDEA*, *DIO2*, and *PPPARGC1A*, *P* < .05 after FDR correction; see Supplementary Fig. S1) (35). Remarkably, when we analyzed the association of those genes with DNL/FA synthesis, *ACLY*, *ACACA*, *ELOVL6*, *FASN*, *MLXIPL*, and *SCD* were correlated with *ACSS2* and *ACSS3*, but *ACSS1* was inversely associated with *SREBF1* and *MLXIPL*, suggesting a different role of the two enzymes in BAT.

### Gene Expression Associations in Low–Brown Adipose Tissue and High–Brown Adipose Tissue Groups—Browning/Thermogenic Markers and β-Oxidation

**Figure 3.**
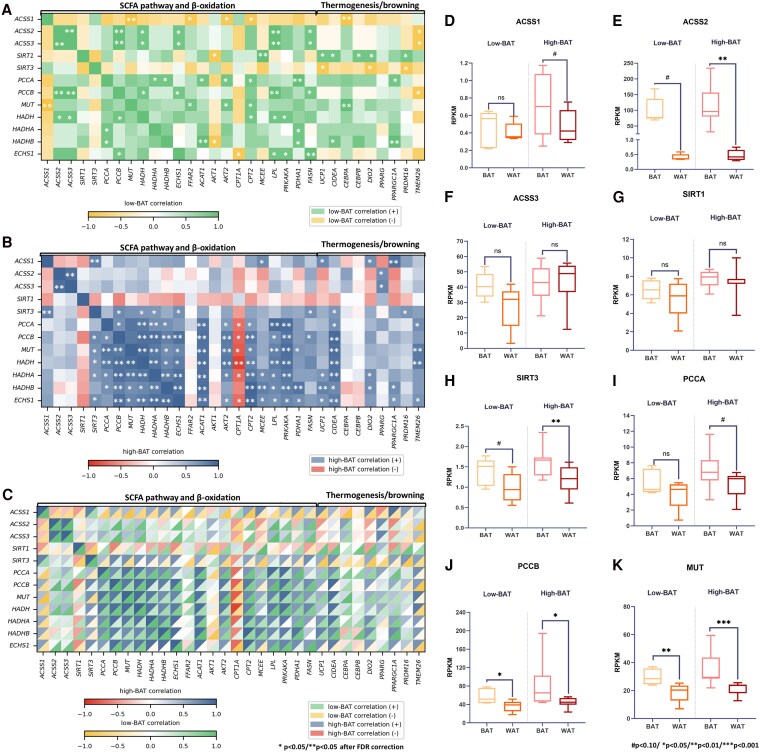
Heat map of correlations between brown adipose tissue (BAT) markers with genes from the short-chain fatty acid (SCFA) pathway and de novo lipogenesis genes according to A, low- (n = 5) and B, high-BAT (n = 9) and C, heat map with split tiles (low-BAT vs high-BAT) and D to K, differences in gene expression in white adipose tissue (WAT) and BAT according to the same groups.

We observed discrepancies between the groups when we investigated how these genes differentially correlated in low-BAT and high-BAT individuals (Fig. 3A and 3B), as highlighted on the split-tile heat map (Fig. 3C). The two groups differed in their associations between *SIRT1*, *SIRT3*, *ACSS1*, and *ACSS2* with SCFA catabolic pathway genes and thermogenic/browning genes (see Fig. 3A-3C). Only in the high-BAT group were positive associations between β-oxidation genes and genes from the SCFA catabolic pathways detected (see Fig. 3B). In the low-BAT group, *PCCA*, *PCCB*, and *MUT*, for example, were not associated with β-oxidation genes, which could suggest different SCFA handling. Moreover, positive associations were observed between *ACSS1* and *SIRT3* in high-BAT volunteers with thermogenic/browning markers (such as *UCP1*, *PRDM16*, *DIO2*, *CIDEA*, and *PPARGC1A*). After FDR correction, some associations between *SIRT3* and *ACSS1* with these markers remained statistically significant. In the low-BAT group, only *SIRT1* was associated with thermogenic markers. Moreover, in the high-BAT group, *SIRT3* was associated with *PCCB*, *ECSH1*, *HADH*, and *HADHA*, all of which were also linked to *CIDEA*. Other genes, such as *HADHA*, *HADHB*, and *ECSH1*, were correlated with *UCP1* and/or *TMEM26* in the high-BAT group but not in the low-BAT group. In contrast, in the low-BAT group, *SIRT3* and *ACSS1* were negatively associated with browning/thermogenic genes (*UCP1*, *PRDM16*, *DIO2*, and *CEBPA*). Using the Fisher r-to-z test, we found that correlations of *SIRT1* and *SIRT3* with thermogenic markers (*UCP1*, *DIO2*, *PRDM16*) differed significantly between high-BAT and low-BAT individuals. In high BAT, associations between SIRT3 were predominantly positive. In contrast, they were weak or negative in low-BAT volunteers, suggesting distinct regulatory interactions between mitochondrial/SCFA pathways and thermogenic gene expression across groups (Supplementary Fig. S2) (35). The opposite was detected for SIRT1 and thermogenic genes (positive associations in the low-BAT group vs negative associations in the high-BAT group).

### Gene Expression Associations in Low–Brown Adipose Tissue and High–Brown Adipose Tissue Groups—De Novo Lipogenesis and Fatty Acid Synthesis

Regarding the associations of SCFA catabolic genes and DNL/FA synthesis genes, the *ACSS2* gene was directly associated with DNL genes in both groups. However, only in the high-BAT group was *ACSS1* inversely correlated with *MLXIPL*. In addition, in the low-BAT group, the *ACSS1* gene was negatively correlated with *AKT2*, *CPT2*, and *MUT* genes (Supplementary Fig. S3) (35). Contrary to the findings from the high-BAT group, positive associations between *ECHS1* with DNL and FA synthesis genes were observed in the low-BAT group, which could suggest that these SCFAs are more easily used for DNL in the BAT of volunteers with higher BAT metabolism (high-BAT group).

### Comparisons Between White Adipose Tissue and Brown Adipose Tissue Gene Expression

We compared the expression of thermogenic, DNL, and SCFA pathway genes in BAT and WAT according to low- and high-BAT groups. The expressions of *PCCB* (Fig. 3I) and *MUT* (Fig. 3J) were shown to be higher in BAT than in WAT for both groups, whereas only in the high-BAT group did we detect higher expressions of *SIRT3* (Fig. 3D) and *ACSS2* (Fig. 3F) and a trend of higher expression of *ACSS1* (Fig. 3E) and *PCCA* (Fig. 3H) compared to WAT. The comparisons between the expression of genes related to SCFA metabolism, β-oxidation, and DNL in BAT vs WAT in the whole sample are depicted in Supplementary Table S3 (35). When comparing low-BAT and high-BAT groups, only *ACSS3* in WAT was significantly higher in the high-BAT group (*P* = .029; data not shown), whereas *SIRT1* reached borderline statistical significance (6.6 ± 1.1 in the low-BAT group vs 7.7 ± 0.9 in the high-BAT group; *P* = .053; data not shown). Even though it would be expected to have higher *ACSS1* and *ACCS2* expression in the BAT of high-BAT individuals, the difference between groups was not statistically significant (*P* = .16; data not shown). Moreover, when comparing groups, no differences were detected for *FFAR2* in WAT or BAT.

**Figure 4.**
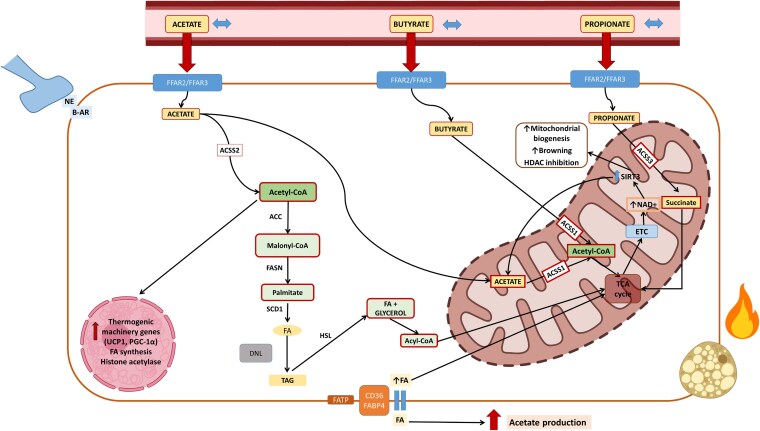
Proposed mechanism linking short-chain fatty acids (SCFAs) metabolism to brown adipose tissue (BAT) function. Cold exposure modulates SCFAs—acetate, butyrate, and propionate—which support BAT thermogenesis. In high-BAT individuals, SCFAs are preserved during cold and correlate with nonesterified fatty acid (NEFA) uptake. On entering brown adipocytes, acetate is converted by *ACSS2* to acetyl-CoA, fueling lipogenesis (via *ACC*, *FASN*, and *SCD1*) and histone acetylation, which promotes thermogenic gene expression (*PGC-1α*). FAs are mobilized (*HSL*) and imported via *CD36*, *FABP4*, and *FATP*. Butyrate enhances mitochondrial biogenesis and browning by inhibiting HDACs and increasing acetyl-CoA via ACSS1. Propionate is metabolized to succinyl-CoA (via *ACSS3*, *PCCA*, *PCCB*, and *MUT*), enters the TCA cycle, and activates *SIRT3* to boost NAD⁺ and ETC activity. Cold and β-adrenergic signaling (NE, β-AR) further promote SCFA uptake and acetate production. Genes involved in SCFA catabolism are enriched in BAT and associate with thermogenesis (*UCP1*, *PRDM16*), β-oxidation (*HADHA/B*, *ECHS1*), and lipogenesis (*FASN*, *ACLY*), linking SCFA availability to metabolic and epigenetic control of browning. β-AR, β-adrenergic receptor; *ACC*, acetyl-CoA carboxylase; *ACSS1/2/3*, acyl-CoA synthetase short-chain family members 1-3; DNL, de novo lipogenesis; ETC, electron transport chain; *FA*, fatty acid; *FASN*, fatty acid synthase; FATP, fatty acid transporters; *FFAR2/3,* free fatty acid receptors 2 and 3; *HDAC*, histone deacetylase; *HSL*, hormone-sensitive lipase; MUT, methylmalonyl-CoA mutase; NE, norepinephrine; *PCCA/PCCB*, propionyl-CoA carboxylase subunits; PGC-1α, peroxisome proliferator–activated receptor γ coactivator 1-α; *SCD1,* stearoyl-CoA desaturase 1; *SIRT3*, sirtuin 3; TAG, triacylglycerol; TCA, tricarboxylic acid cycle; *UCP1*, uncoupling protein 1. (Created in BioRender. Pires, M. (2025) https://BioRender.com/js8rqie).

Lastly, Fig. 4 depicts the proposed mechanisms by which circulating SCFAs may function as markers of proper BAT function, especially concerning the uptake of FAs during cold exposure.

## Discussion

Compelling evidence highlights the role of microbiota-derived metabolites in energy balance and adipose tissue function. Yet, research on the crosstalk between circulating SCFAs and BAT function in humans is limited. Our study aimed to explore the relationship between circulating SCFAs and BAT metabolism in adults. CI circulating acetate was associated with CI-NEFA uptake in BAT, while CI-propionate correlated with CI-BAT MRO_2_. These results support the idea that circulating SCFAs may be valuable markers for proper BAT function. Supporting these findings, individuals in the high-BAT group had higher CI, but not RT, SCFA levels. In BAT samples from this group, thermogenic genes like *UCP1*, *CIDEA*, and *PRDM16* correlated with genes involved in SCFA catabolism. Additionally, propionate-related genes (*PCCA*, *PCCB*, and *MUT*) were linked to β-oxidation markers, reinforcing the potential of SCFAs to indicate BAT function and facilitate FA uptake.

Several rodent studies have demonstrated SCFAs’ role in adipose tissue by modulating lipolysis, lipogenesis, and thermogenesis (3, 6, 10, 15). SCFAs serve as lipid synthesis substrates and signaling molecules that regulate adipose tissue metabolism (6, 24, 37). Acetate and butyrate are readily converted into acetyl-CoA, a precursor for FA and cholesterol synthesis (3), fuel for BAT and WAT under nutrient deprivation (38, 39), or HDAC inhibitors affecting gene transcription (14, 38, 40). Still, acetate's direct effects in brown adipocytes remain contradictory (41, 42). Our study showed associations between CI-acetate, circulating FFAs, and CI-NEFA uptake in BAT, suggesting that acetate could be a marker of FFA uptake in BAT. Notably, this correlation was present only in individuals in the high-BAT group. No associations were seen between CI-SCFAs and CI-glucose uptake, which aligns with the understanding that BAT relies primarily on TG-derived FAs over glucose during its activation, with SCFAs supporting this function either as energy sources or HDAC inhibitors (6, 12, 24, 37, 43). Moreover, BAT can use intracellular and extracellular lipids during cold exposure (44).

The metabolic relevance of SCFAs has also been linked to their contribution to the acetyl-CoA pool (9, 38). Current evidence suggests that acetate and acetyl-CoA levels may function as metabolic sensors, connecting nutrient availability to stress responses and gene regulation (38, 39). Elevated intracellular acetyl-CoA modulates histone acetylation and thus metabolic gene expression (24, 38, 39). While butyrate more strongly influences histone acetylation (24, 45), we observed no correlation between circulating butyrate and BAT function. This may result from rapid butyrate conversion to acetyl-CoA or its use in histone acetylation during thermogenesis (38, 39). Contrary to this idea, one in vitro study reported that acetate inhibited thermogenic machinery in brown adipocytes, reducing mitochondrial uncoupling and FA oxidation (42).

Interestingly, we also detected a strong correlation between CI-propionate and the CI-MRO_2_ in BAT, observed only in the low-BAT group after stratifying the sample. In vitro evidence suggests that propionate activates the sympathetic nervous system, increasing heart rate, thermogenesis, and energy expenditure (46), and induces WAT browning (24, 46). The absence of a similar correlation in the high-BAT group may reflect distinct mechanisms of BAT fuel utilization and activation across groups. Although somewhat paradoxical, the correlation between CI-propionate and MRO_2_ in the low-BAT group might indicate increased sympathetic activity or muscle shivering, potentially explaining the observed serum propionate reductions after cold exposure. In contrast, individuals with high-BAT activity may possess more efficient metabolic machinery to use SCFAs, whether as oxidative fuel, substrates for DNL, or regulators of gene transcription via histone acetylation (24, 25, 38, 39).

Stratification of the sample also revealed distinct CI-SCFA patterns: While SCFA levels remained stable after cold in the high-BAT group, propionate and butyrate concentrations decreased in the low-BAT group. Since SCFAs can act as nutrient sensors, the stable levels in high-BAT individuals may reflect enhanced regulation of SCFA production or utilization (possibly hepatic) to sustain thermogenesis (25, 38). Cold exposure activates gut-brain communication via transient receptor potential channels, stimulating norepinephrine release and BAT thermogenesis, while promoting WAT browning (47). In the low-BAT group, the SCFA reduction may suggest preferential use of SCFAs as readily absorbed and metabolized oxidative substrates for thermogenesis. Yet, the specific mechanisms remain uncertain. Indeed, CI SCFA alterations are not well defined in humans (36, 48). This means that, during cold exposure, high-BAT individuals maintain stable levels of circulating acetate and propionate—associated with BAT FA uptake —while low-BAT individuals show a drop in these SCFAs, suggesting that SCFA stability supports efficient thermogenesis and substrate handling under cold stress. However, because the effects of cold on SCFA production are controversial and not well explored in humans (36, 48), intervention studies are necessary to confirm this hypothesis. A 10-day cold challenge study in mice reported impaired gut microbiota and diminished SCFA levels (36), though other studies have shown variable responses depending on exposure duration and intensity (36, 48, 49).

Remarkably, the absence of gut microbiota reduces rodent thermogenic capacity—a defect reversed by butyrate supplementation (48). Elevated circulating butyrate may confer a metabolic advantage by boosting mitochondrial activity and FA oxidation in BAT, as shown in mice (6). Both butyrate and acetate also promote WAT browning by upregulating genes such as *UCP1*, *PRDM16*, and *PGC-1α*, leading to browning (15, 21, 23). Additionally, SCFAs—particularly acetate and butyrate—act as potent HDAC inhibitors, enhancing histone acetylation and transcription of genes involved in cell differentiation, metabolism, and anti-inflammatory pathways (15, 24). In adipose tissue, HDAC3 inhibition has been linked, for example, to increased expression of PPARγ targets, improving insulin sensitivity (24). Nevertheless, the cell- and gene-specific pathways of SCFA-mediated HDAC inhibition remain only partially understood.

We also observed positive associations between serum RT and CI-acetate and propionate with the insulin sensitivity marker (M-value) and HDL cholesterol, alongside inverse correlations between RT-acetate and body weight, BMI, and waist circumference. These findings support previous associations between higher circulating SCFAs and improved lipid and glucose metabolism (2, 11, 50, 51). SCFAs play an essential role in glucose regulation by acting on GPCRs, especially *FFAR2* (GPR43), which is expressed in enteroendocrine cells and pancreatic islets (2). *FFAR2* activation stimulates glucagon-like peptide-1 release, enhancing insulin secretion and suppressing glucagon, improving postprandial glucose control (6, 24). SCFAs also regulate energy metabolism through AMPK signaling (6, 21, 22), promoting mitochondrial biogenesis and glucose uptake via PGC-1α, particularly in muscle and adipose tissue (6). Unexpectedly, we detected no differences in *FFAR2* expression between low- and high-BAT groups. Interestingly, most SCFA-clinical parameter associations were detected only in the high-BAT group, emphasizing the relevance of elevated circulating SCFA. We hypothesize that acetate may be converted into acetyl-CoA for DNL, with newly synthesized FAs possibly activating UCP1 instead of being stored as TGs. Indeed, our transcriptome results linking the SCFAs catabolic pathway and thermogenic genes in BAT samples add substantial insights to this narrative.

In high-BAT individuals, *ACSS1* positively correlated with *SIRT3*, *PGC1-α*, and *UCP1*, and *SIRT3* with all β-oxidation genes, indicating robust thermogenic capacity under cold exposure. SCFA, mainly acetate, is converted to acetyl-CoA by *ACSS1*, supporting energy homeostasis and mitochondrial function during fasting or ketogenic states (38, 52). This means *ACSS1* provides an alternative route for generating acetyl-CoA from SCFAs, mainly when nutrient availability is limited or during thermogenic events (38, 52), and may influence epigenetic regulation via SIRT3-mediated deacetylation (53, 54). Mice lacking *ACSS1* develop hypothermia during fasting or under low-carbohydrate diets, while wild-type mice increase *ACSS1* expression under similar conditions (38, 55). Experimental data support the hypothesis that cold exposure and caloric restriction activate *SIRT3* expression in WAT and BAT (53), and *SIRT3* knockout mice exhibit impaired lipid metabolism (56). Thus, in our samples, *SIRT3* and *ACSS1* seem to support the metabolic needs of BAT, ensuring that energy can be produced efficiently, even when substrate availability is scarce (53, 57), like using SCFA during cold exposure.

We also found correlations between *CIDEA* and *TMEM26* with SCFA-pathway catabolic genes in the high-BAT group, especially those in propionate metabolism. *CIDEA* was associated with *PCCA*, *PCCB*, and *MUT*, which are involved in propionate catabolism. *CIDEA* regulates lipid droplet dynamics, controlling FA availability for β-oxidation (58, 59), and supports energy balance during cold or fasting (58, 59). By controlling lipid droplet formation and turnover, *CIDEA* modulates the substrates accessible to β-oxidation enzymes (58, 59). This regulation is essential for maintaining the energy balance within BAT, particularly during periods of increased energy demand, such as cold exposure or fasting (58, 59). On the other hand, by deacetylating key enzymes involved in β-oxidation, *SIRT3* directly enhances their metabolic efficiency, counterbalancing the lipid storage function of *CIDEA* (54, 56, 57, 60). The interconnected roles of *CIDEA*, *SIRT3*, and *ACSS1* in our high-BAT volunteers suggest a finely tuned regulatory network within BAT that balances energy storage and expenditure, maintaining metabolic flexibility required for effective thermogenesis, and allowing BAT to respond dynamically to physiological. Conversely, the low-BAT group showed negative associations of *SIRT3* with *UCP1*, and *ACSS1* with *MUT* and *FFAR2*, hinting at a less efficient thermogenic system. Recent findings show that adding propionate to mature adipocytes increases *UCP1* and *PGC1α*, especially from mesenteric fat, enhancing mitochondrial respiration and adenosine triphosphate output (61).

In high-BAT individuals, *ACSS2* expression also correlated with DNL genes like *ACACA* and *ACLY*, suggesting a coordinated balance between lipolysis and lipogenesis. Mice exposed to mild cold increase DNL gene expression via the AKT2-ChREBP pathway, which stimulates *ACSS2* to convert acetate into acetyl-CoA for BAT utilization (62). ACSS2 also recycles acetate from nuclear deacetylation reactions, such as HDAC activity (63-65) and can bind acetylated PPARγ, recruiting *SIRT1* and *PRDM16* to activate UCP1 transcription (65).

When comparing BAT and WAT depots, differences in the expression of *ACSS1*, *ACSS2*, and *SIRT3* were observed only in the high-BAT group, suggesting a more metabolically flexible BAT capable of efficient substrate utilization. FAs not only fuel thermogenesis but also contribute to its activation in BAT (43, 44, 66). Interestingly, cold exposure simultaneously induces β-oxidation and lipogenesis in BAT—two typically opposing pathways—highlighting BAT's unique metabolic coordination (44). In our study, high-BAT individuals displayed a more orchestrated metabolic profile linking SCFA signaling with lipolysis and lipogenesis, as observed from circulating SCFA associations and gene expression analysis. We also found correlations between SCFA pathway genes and *AKT2* in high-BAT individuals and *Elovl6* in both groups. *AKT2* is a cold-inducible kinase that promotes ChREBP-mediated DNL, supplying FAs essential for mitochondrial heat production (62), whereas Elovl6 influences long-chain FA composition, regulating mitochondrial efficiency and thermogenic adaptation. Elovl6 deficiency impairs lipid oxidation and cold response in BAT, emphasizing its role in fuel management (66). These insights underscore the complexity of metabolic regulation in BAT and suggest that modulating lipid-derived signals and metabolic enzymes may offer therapeutic potential for metabolic disorders.

Our study does have limitations. Its cross-sectional design prevents causal inference, and differences between the two cohorts (such as in age and BMI) may have affected BAT metabolic responses. While the sample size for gene expression analysis (n = 14) is modest, it remains acceptable for human studies, and most blood markers and imaging data were collected from more individuals. The lack of dietary and microbiota data limits our conclusions on gut-derived SCFA production. Nonetheless, using four PET/CT radiotracers allowed a multidimensional assessment of BAT metabolism—including glucose and NEFA uptake, perfusion, and MRO_2_—in relation to serum SCFAs. Although most of the genes are not exclusively involved in SCFA pathways, the integration of imaging data with serum SCFA levels and transcriptomic correlations suggests that these genes may act in concert to orchestrate SCFA handling and utilization within metabolically active BAT.

Our findings suggest that circulating SCFAs are not only markers but potential modulators of BAT function, especially under cold stress. During cold exposure, high-BAT individuals maintained higher levels of circulating SCFAs while low-BAT individuals exhibited a drop in these metabolites. These differences were absent at RT, suggesting a specific SCFA resilience mechanism during thermal stress. Moreover, circulatory acetate levels correlated with BAT FA uptake, and propionate with oxygen consumption, implying that stable SCFA levels support thermogenic substrate handling. These results support the hypothesis that maintaining SCFA availability—possibly through dietary fiber or microbiota-targeted interventions—may enhance BAT thermogenesis and systemic metabolic flexibility. Future clinical trials aimed at increasing endogenous SCFA production could help clarify the therapeutic potential of SCFA modulation in promoting BAT activity and metabolic health.

## Data Availability

The data that support the findings of this study are available from the corresponding authors on reasonable request and will be provided within a reasonable time frame.

## Abbreviations

BAT: brown adipose tissue
BMI: body mass index
CI: cold-induced
CT: computed tomography
DNL: de novo lipogenesis
FDR: false discovery rate
GPCRs: G protein–coupled receptors
GUR: glucose uptake rate
HDACs: histone deacetylases
HDL: high-density lipoprotein
LDL: low-density lipoprotein
MRO_2_: metabolic rate of oxygen
NEFA: nonesterified fatty acid
PET: positron emission tomography
PPAR-γ: peroxisome proliferator–activated receptor γ
RT: room temperature
SCFA: short-chain fatty acid
SUV: standardized uptake value
TCA: tricarboxylic acid cycle
TGs: triglycerides
WAT: white adipose tissue
